# IMD-4690, a Novel Specific Inhibitor for Plasminogen Activator Inhibitor-1, Reduces Allergic Airway Remodeling in a Mouse Model of Chronic Asthma via Regulating Angiogenesis and Remodeling-Related Mediators

**DOI:** 10.1371/journal.pone.0121615

**Published:** 2015-03-18

**Authors:** Toshifumi Tezuka, Hirohisa Ogawa, Masahiko Azuma, Hisatsugu Goto, Hisanori Uehara, Yoshinori Aono, Masaki Hanibuchi, Yoichi Yamaguchi, Tomoyuki Fujikawa, Akiko Itai, Yasuhiko Nishioka

**Affiliations:** 1 Department of Respiratory Medicine and Rheumatology, Institute of Health Biosciences, University of Tokushima Graduate School, Tokushima, Japan; 2 Department of Molecular and Environmental Pathology, Institute of Health Biosciences, University of Tokushima Graduate School, Tokushima, Japan; 3 Institute of Medicinal Molecular Design Inc., Tokyo, Japan; Medical University of South Carolina, UNITED STATES

## Abstract

Plasminogen activator inhibitor (PAI)-1 is the principal inhibitor of plasminogen activators, and is responsible for the degradation of fibrin and extracellular matrix. IMD-4690 is a newly synthesized inhibitor for PAI-1, whereas the effect on allergic airway inflammation and remodeling is still unclear. We examined the *in vivo* effects by using a chronic allergen exposure model of bronchial asthma in mice. The model was generated by an immune challenge for 8 weeks with house dust mite antigen, *Dermatophagoides pteronyssinus* (Dp). IMD-4690 was intraperitoneally administered during the challenge. Lung histopathology, hyperresponsiveness and the concentrations of mediators in lung homogenates were analyzed. The amount of active PAI-1 in the lungs was increased in mice treated with Dp. Administration with IMD-4690 reduced an active/total PAI-1 ratio. IMD-4690 also reduced the number of bronchial eosinophils in accordance with the decreased expressions of Th2 cytokines in the lung homogenates. Airway remodeling was inhibited by reducing subepithelial collagen deposition, smooth muscle hypertrophy, and angiogenesis. The effects of IMD-4690 were partly mediated by the regulation of TGF-β, HGF and matrix metalloproteinase. These results suggest that PAI-1 plays crucial roles in airway inflammation and remodeling, and IMD-4690, a specific PAI-1 inhibitor, may have therapeutic potential for patients with refractory asthma due to airway remodeling.

## Introduction

Bronchial asthma is characterized by allergic inflammation, airway hyperresponsiveness (AHR), and remodeling, including epithelial injury, subepithelial thickening/fibrosis, extracellular matrix (ECM) deposition, airway smooth muscle hyperplasia, goblet cell hypertrophy and hyperplasia, and angiogenesis [1].

Recent studies suggest that the fibrinolytic system plays a key role in the development of airway remodeling. Plasmin, the key enzyme of fibrinolysis, is derived from plasminogen through the catalytic action of plasminogen activators (PAs), tissue-type PA (tPA) and urokinase-type PA (uPA) [2]. The tPA-mediated plasminogen activation plays a main role in the dissolution of fibrin in the circulation. On the other hand, uPA binds to a specific cellular receptor (uPAR), resulting in enhanced activation of cell-bound plasminogen [3]. Plasmin can degrade fibrin and activate the matrix metalloproteinase (MMP) system, which is involved in degrading ECM proteins (such as collagen) and neutralized by tissue inhibitors of metalloproteinase (TIMP) [4]. Recently, it was shown that enhancement of uPA/Plasmin activity reduces airway remodeling in a murine asthma model [5].

Among the plasminogen activator inhibitors (PAIs), PAI-1 is the principal inhibitor of PAs [4]. Mast cells are an important source of PAI-1 in the asthmatic airway [6], and elevated plasma levels of PAI-1 are associated with poor lung function in asthmatic patients [7]. PAI-1 is the main inhibitor of MMPs, and the major MMP released in the airway of asthmatics is MMP-9, which is mainly produced by alveolar macrophages [8]. Compared with the wild-type (WT) mice, in PAI-1-deficient mice, collagen and fibrin depositions were less in the lung tissue and MMP-9 activity was higher in both lung tissues and bronchoalveolar lavage fluid (BALF) after OVA challenge; this finding indicated that a lack of PAI-1 may prevent collagen deposition by MMP-9 activity in the asthmatic airway [9].

PAI-1 may also contribute to airway remodeling by regulating vascular endothelial growth factor (VEGF). VEGF induced T-helper type 2 cell (Th2)-mediated inflammation and airway remodeling and anti-VEGF receptor antibodies reduced eosinophil infiltration in a murine model [10, 11]. In PAI-1 deficient mice, the VEGF expression was significantly reduced compared with control mice [12].

We, therefore, examined whether a specific PAI-1 inhibitor, IMD-4690, affected airway inflammation, AHR, and airway remodeling, including subepithelial fibrosis, smooth muscle cell hypertrophy and angiogenesis, in a chronic antigen exposure model of asthma in mice.

## Materials and Methods

### Molecular design and synthesis of IMD-4690

A synthetic PAI-1 inhibitor, IMD-4690, 2-[[3-(4-tert-Butylphenoxy)-4'-(trifluoromethoxy) [1,1'-biphenyl]-4-yl]oxy] acetic acid, was molecularly designed, synthesized, and provided by the Institute of Medicinal Molecular Design Inc. (Tokyo, Japan). IMD-4690 powder was dissolved in 0.5% carboxymethylcellulose (CMC; Sigma-Aldrich Japan, Tokyo, Japan).

### Inhibition of PAI-1 by IMD-4690

Inhibitory effect of IMD-4690 on the activity of PAI-1 was measured by the direct tPA assay. Briefly, recombinant tPA and PAI-1 was mixed with IMD-4690, and then tPA substrate with fluorescent pigment (Pyr-Gly-Arg-MCA) was added in this mixture and incubated. The enzymatic activity was calculated by measuring the fluorescence. The inhibitory activity of IMD-4690 on other enzymes was measured with the similar method.

### Preparation of house dust mite antigen

House dust mite antigen (*Dermatophagoides pteronyssinus* [Dp]) was purchased from LSL (Tokyo, Japan). This extract included major allergens, Der p 1 and Der p 2, and was proteolytically active [13]. Endotoxin removal solution (Sigma-Aldrich Japan) was used to reduce the endotoxin concentration. After removal, Dp endotoxin was 0.308 IU/mg.

### Mouse experimental protocols

Six-week-old female BALB/c mice (15–20 g) were purchased from CLEA Japan Inc. (Tokyo, Japan). Mice were maintained in the animal facility of the University of Tokushima according to the guidelines of the ethics committee of our university [14]. The present study was approved by Institutional Animal Care and Use Committee of the University of Tokushima (Permission Number: 11134). Mice were housed under specific pathogen-free conditions with 12-h light/dark cycle in a temperature-controlled room with free access to food and water. Mice were monitored weekly for body weight and daily for food and water intake and assessed visually for signs of clinical disease including inactivity, labored respiration and ruffled fur. Mice that lost more than 30% of their original body weight and/or displayed evidence of severe asthma attack were euthanized by overdose of intraperitoneal injection of 2,2,2-tribromoethanol (Sigma) with 2-methyl-2-butanol (Sigma), and all efforts were made to minimize suffering [14].

**Fig 1:**
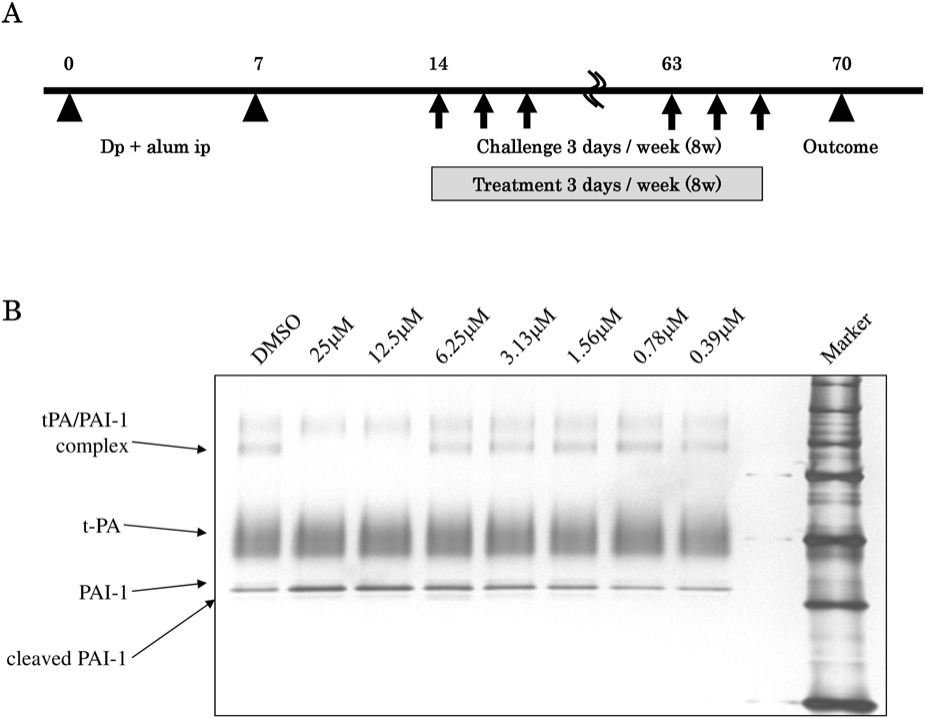
Schedule of *in vivo* experiments and biological activity of IMD-4690. (A) Protocols for the mouse experiments. (B) Inhibitory effects of IMD-4690 on the binding of tPA and PAI-1. The recombinant PAI-1 and IMD-4690 were mixed and incubated in PBS at 37°C for 5 min. Then tPA was added. After incubation at 37°C for 5 min, samples were applied for 10% SDS-PAGE and visualized with a silver staining. The bands of tPA/PAI-1 complex were quantified by a densitometry, and calculated IC50.

All mice were sensitized on days 0 and 7 by intraperitoneal injections of 10 μg of Dp dissolved in 500 μL saline and mixed with 1mg of Alum (Sigma-Aldrich Japan). Dp-sensitized mice were challenged intranasally with 10 μg (10 μL) Dp in 70 μL saline on alternate days, 3 days per week, from days 14 to 67. The mice were also treated intraperitoneally with IMD-4690 (10 mg/kg) (Dp/IMD group) or CMC alone (Dp/CMC group) at the same day with Dp challenge. As a control, mice were challenged with saline and given CMC during this period (control/CMC group) (Fig. 1A). Each experiment was performed using six mice per group.

### Measurement of lung resistance

Seventy-two hours after the final challenge (day 70), lung resistance (R_L_) was measured by restrained whole body plethysmography (Buxco Electronics). Mice were anesthetized by 6 mg/mL 2,2,2-tribromoethanol with 2-methyl-2-butanol. Then mice were tracheostomized and cannulated with a 19-gauge tracheostomy tube, and were placed in the chamber for plethysmography and mechanically ventilated. Aerosolized β-methacholine (Mch) was administered by an in-line aerosol delivery system at different concentrations (6.25, 12.5, 25, and 50 mg/mL). After each Mch challenge, data were continuously collected, and values of R_L_ were taken to express the changes in these functional parameters. The data were expressed as the percentage change from baseline R_L_ obtained after inhalation of saline.

### Bronchoalveolar lavage

After measurement of lung resistance, the mice were euthanized humanely by cutting the axillary artery to obtain serum samples. Following sacrifice, bronchoalveolar lavage was performed twice with saline (0.8 mL) by using a soft cannula. After counting the cell numbers in the BALF using the automated cell viability analyzer Vi-Cell (Beckman Coulter, Calif., USA), BALF samples were cytospun onto glass slides and stained with Diff-Quick (Baxter, Miami, Fla., USA) for classification.

### Histopathology

The lung tissues were harvested, fixed in 10% formalin, and embedded in paraffin. Three-micrometer-thick sections were stained with Giemsa, Periodic acid-Schiff (PAS) or Azan Mallory.

Sections of lung tissue were stained immunohistochemically to detect α-smooth muscle actin (SMA), platelet endothelial cell adhesion molecule-1 (PECAM-1)/CD31. Mouse monoclonal anti-smooth muscle (1:800) (Thermo Fisher Scientific Inc., Fremont, CA, USA), or ready-to-use mouse monoclonal CD31 (GeneTex Inc., Irvine, CA, USA) was used as a primary antibody. For immunohistochemical staining, the VECTASTAIN ABC Kit (Vector Laboratories) and the Vector M.O.M. Immunodetection Kit (Vector Laboratories) were used according to the manufacturers’ protocols. Diaminobenzidine was used as a substrate for the immunoperoxidase reaction. Sections were lightly counterstained with hematoxylin and analyzed by bright-field microscopy.

### Morphological analyses

An OLYMPUS BX61 microscope (Olympus, Tokyo, Japan) with Scion Image software was used for the morphological analysis. Eosinophils were counted according to a previously described method [15]. The thickness of subepithelial fibrosis and smooth muscle layer were analyzed as follows. The area of subepithelial fibrosis (stained blue) and smooth muscle layer (α-SMA positive) around a bronchus was measured. The average thickness was determined by the area of the positive layer divided by the length of the internal circumference of the area. The mean values for thickness were calculated for 8–10 bronchi per left lung lobe.

To quantify the microvessel density, the number of CD31-positive blood vessels per field was counted in 5 random fields in each sample.

### Homogenization of whole lungs

The right lungs were frozen until analyzed. Frozen tissue were solubilized by homogenization in lysis buffer (Cell Signaling Technology, Inc., Danvers, Mass., USA) containing 1 tablet of Complete Protease Inhibitor Cocktail Tablets, EDTA-free (Santa Cruz Biotechnology, Inc., Santa Cruz, CA, USA), per 25 mL of buffer by using an Ultramicro homogenizer set (I.S.O.,Inc., Yokohama, Japan). Samples of the homogenate were centrifuged at 15,000 rpm for 10 min. The supernatants were stored at -80°C.

### Collagen assay

The left lungs were harvested on day 70 and used for collagen assays. Total lung collagen was determined using a Sircol Collagen Assay kit (Biocolor Ltd, Belfast, Northern Ireland) according to the manufacturer’s instructions [16, 17].

### Measurements of total protein and cytokine

Protein concentrations were determined by the BCA method (Pierce, Rockford, IL, USA). Cytokines, growth factors, enzymes, and serum IgE were determined using commercial ELISA kits. ELISA kits and their sensitivities were as follows: IL-4, -5, -12, -13, TNF-α, IFN-γ, VEGF, TGF-β, total MMP-9, and TIMP-1 (R&D Systems, Minneapolis, MN, USA), with sensitivities of 2, 7, 2.5, 1.5, 5.1, 2, 3, 1.7, 7, and 1.4 pg/mL, respectively; active and total PAI-1 (Innovative Research Inc., Sarasota, FL, USA) with sensitivities of 20 and 32 pg/mL; active MMP-9 (MMP-9 Biotrak Activity Assay, GE Healthcare, Buckinghamshire, UK) with a sensitivity of 0.5 ng/mL; HGF (Rat HGF determination kit by EIA, Institute of Immunology Co., Ltd., Tokyo, Japan) with a sensitivity of 0.4 ng/mL; and serum IgE (ELISA mouse IgE kit, Seikagaku Biobussiness Corp., Tokyo, Japan) with a sensitivity of 0.5 ng/mL.

### Statistical analysis

Data analysis was performed using GraphPad Prism, version 5.0 (GraphPad software, San Diego, CA). Experimental results were expressed as means ± SE. Experimental group results were compared using one-way ANOVA. If statistical significance was identified by ANOVA, we used a Tukey–Kramer test to correct for multiple comparisons. Differences were considered to be statistically significant if p values were less than 0.05.

## Results

### Inhibitory effects of IMD-4690 on the activity of PAI-1 and the binding of tPA and PAI-1 *in vitro*

A synthetic PAI-1 inhibitor, IMD-4690, 2-[[3-(4-tert-Butylphenoxy)-4'- (trifluoromethoxy) [1,1'-biphenyl]-4-yl]oxy] acetic acid, was molecularly designed, synthesized. IMD-4690 inhibited the binding of tPA and PAI-1 at the IC50 of 8 μM (Fig. 1B). In addition, IMD-4690 specifically inhibited the biological activity of PAI-1 (IC50 = 1.1 μM), but not PAI-2 and-3, anti-thrombin 3, α1-anti-trypsin, α1-anti-chymotripsin, α1-anti-plasmin and Heparin cofactor II (Table 1).

**Table 1:** Human SERPIN inhibition selectivity of IMD-4690.

SERPIN	Ser Protease	Inhibition IC50 (μM)
PAI-2 (SERPINB2)	uPA	>25
PAI-3 (Protein C inhibitor/SERPINA5)	uPA	>25
Anti-thrombin (AT3/SERPINC1)	Thrombin factor Xa	>25
α1-anti-trypsin (SERPINA1)	Trypsin Neu-Elastase	>25
α1-anti-chymotrypsin (SERPINA3)	Chymotrypsin Cathepsin G	>25
α1-anti-plasmin (SERPINF2)	Plasmin	>25
Heparin Cofactor 2 (HC2/SERPIND1)	Thrombin	>25

IC50 of human PAI-1 inhibition: 1.1 μM

### Inhibition of active PAI-1 by IMD-4690 in chronic allergen exposure model of bronchial asthma in mice

**Fig 2:**
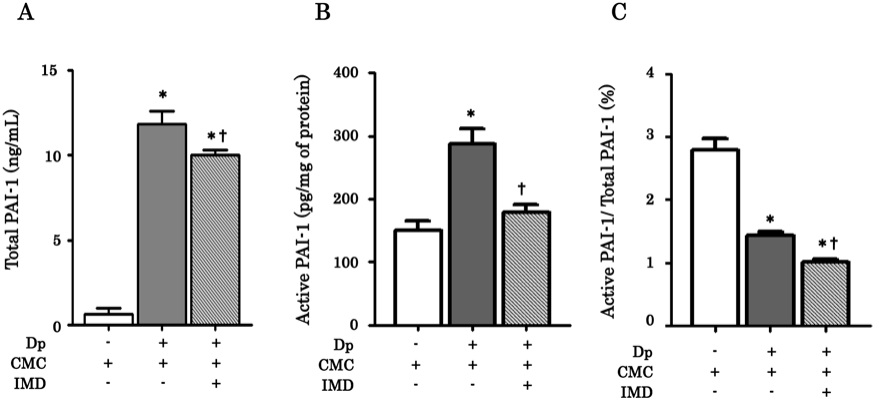
Inhibition of active PAI-1 levels by IMD-4690 in mice. (A) total PAI-1 in lung homogenates, (B) active PAI-1 in lung homogenates, (B) The ratio of active PAI-1 to total PAI-1 concentration in lung homogenates. Data are expressed as means ± SE of six mice. *P < 0.05 compared with control/CMC mice, ^†^P < 0.05 compared with Dp/CMC mice.

Next we examined the effects of IMD-4690 on chronic allergen exposure model of bronchial asthma in mice. We first confirmed the activity of IMD-4690 for the level of PAI-1 in lung homogenates. The level of total and active PAI-1 was increased in Dp/CMC mice compared with control/CMC mice in the lung homogenates (Fig. 2A and 2B). Treatment with IMD-4690 significantly reduced the level of both total and active PAI-1 (Fig. 2A and 2B). In addition, the proportion of active PAI-1 as a percentage of the total PAI-1 in Dp/IMD mice was significantly lower than that in Dp/CMC mice (Fig. 2C). These results suggest that prolonged administration of IMD-4690 inhibits PAI-1 production as well as activation in the lungs of a mouse model of chronic asthma.

**Fig 3:**
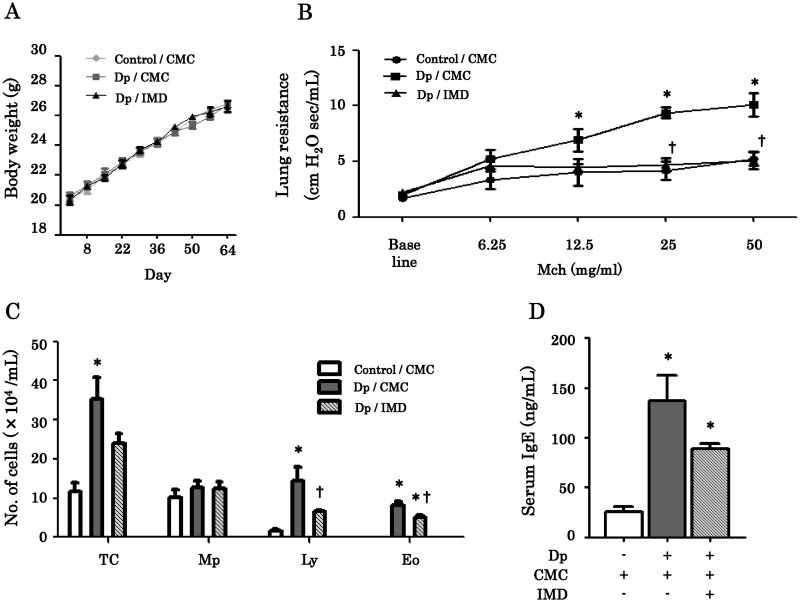
Changes in body weight and effects of IMD-4690 in the allergic airway inflammation model of mice. (A) Body weight measures in chronic exposure mice. (B) Assessment of airway resistance after Dp challenge. (C) BALF cell analysis. TC, total cells; Mp, macrophage; Ly, lymphocytes; Eo, eosinophils. (C) Serum IgE. Data was analyzed by ELISA. Data are expressed as means ± SE of six mice. *P < 0.05 compared with control/CMC mice, ^†^P < 0.05 compared with Dp/CMC mice.

When we examined the changes in body weight, treatment with IMD-4690 did not show any weight loss for Dp-sensitized mice, indicating the less toxicity (Fig. 3A).

### Effect of IMD-4690 on AHR in chronic allergen exposure model of bronchial asthma in mice

To examine the effects of IMD-4690 on AHR in the chronic model, we assessed R_L_ by restrained plethysmography. Repeated-measures two-way ANOVA showed that the curves for all groups were significantly different (F(8, 75) = 3.32, P = 0.0027). The R_L_ was significantly increased in Dp/CMC mice at 12.5, 25, and 50 mg/mL of Mch. IMD-4690 significantly reduced airway resistance compared to that of Dp/CMC mice at 25 and 50 mg/mL of Mch (Fig. 3B). These findings suggest that administration of IMD-4690 improved AHR in chronic allergen exposure model of bronchial asthma in mice.

### Analysis of cell classification of BALF and serum IgE

We next assessed airway inflammation using BALF. BALF samples were collected and cell classifications were performed as described in Materials and Methods. The total cell counts in the BALF recovered from Dp/CMC mice were significantly higher than the cells in the BALF samples from the control/CMC mice (Fig. 3C). In particular, lymphocytes and eosinophils counts increased in Dp/CMC mice. On the other hand, treatment with IMD-4690 significantly decreased the numbers of lymphocytes and eosinophils, as compared with Dp/CMC mice.

Dp exposure induced a significant increase in the serum levels of IgE in Dp/CMC and Dp/IMD mice compared with those of control mice. As shown in Fig. 3D, treatment with IMD-4690 significantly decreased serum IgE.

### Effects of IMD-4690 on eosinophil infiltration and the level of related cytokines in chronic allergen exposure model of bronchial asthma in mice

**Fig 4:**
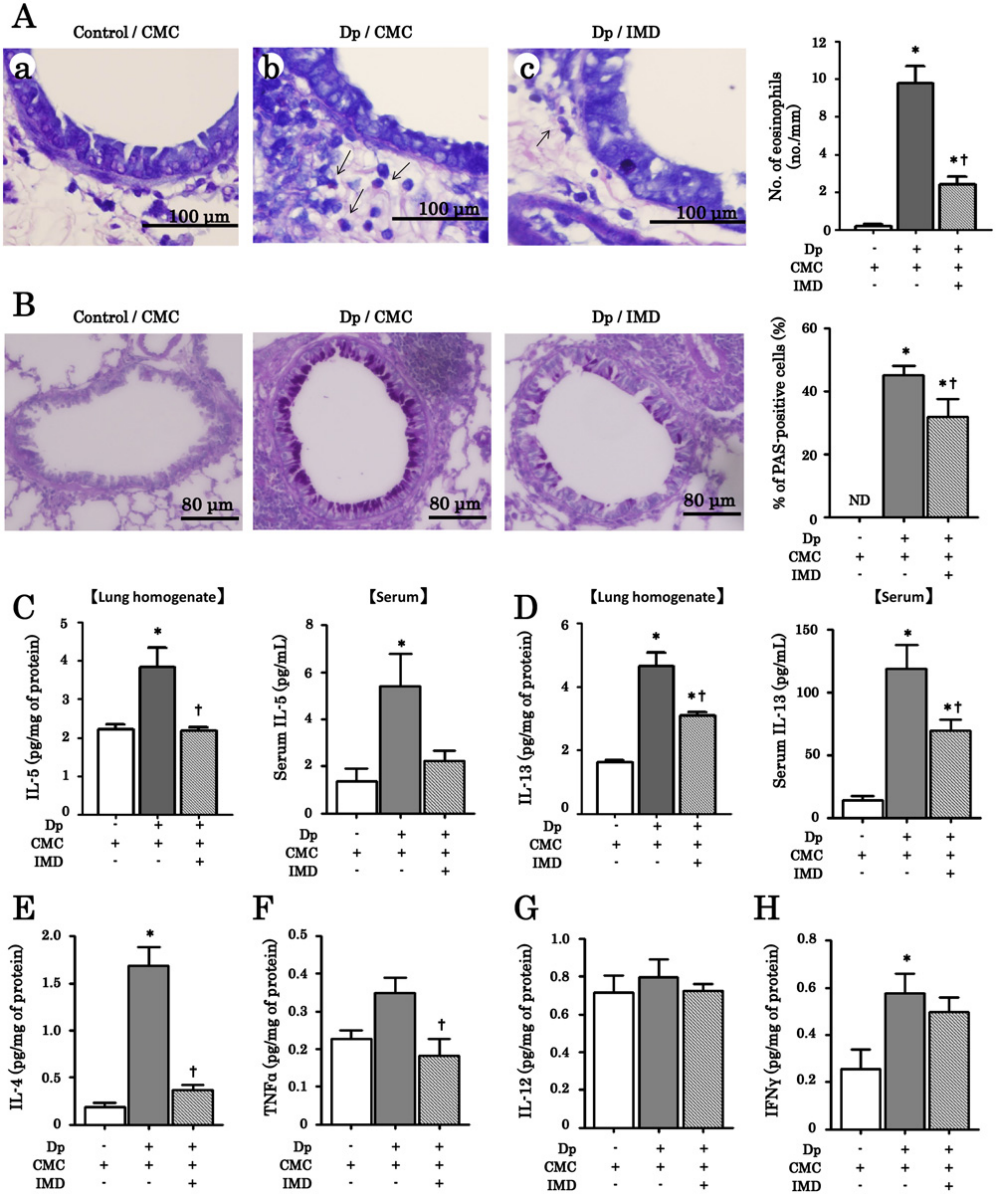
Anti-allergic effects of IMD-4690 in the chronic asthma model of mice. (A) Histopathology (Giemasa stain) in the lungs. Arrowheads indicate eosinophils. Original magnification in all figures: ×400. Scale bars in all figures = 100 μm. The number of eosinophils in the subepithelium is also counted and expressed as a graph. (B) PAS staining in the lungs. The PAS-positive cells indicate mucous metaplasia. The number of PAS-positive cells is counted and expressed as a graph. (C-H) Cytokine levels in mouse lung homogenates (C-H) and serum (C, D). IL-5 (C), IL-13 (D), IL-4 (D), TNF-α (E), IL-12 (F) and IFN-γ were measured by ELISA. Data are expressed as means ± SE of six mice. *P < 0.05 compared with control/CMC mice, ^†^P < 0.05 compared with Dp/CMC mice.

In the Giemsa-stained sections, the number of infiltrating eosinophils in the subepithelium increased in Dp/CMC mice compared to control/CMC mice (Fig. 4A). Treatment with IMD-4690 significantly decreased the numbers of infiltrating eosinophils (Fig. 4A). The Periodic acid-Schiff (PAS) staining also showed mucous metaplasia in airway epithelial cells in Dp/CMC mice, and the number of PAS-positive cells was decreased by treatment with IMD-4690 (Fig. 4B). We also examined cytokine concentrations in lung homogenates, as some cytokines were not detected in the BALF. Th2-cytokines including IL-4, IL-5 and IL-13 levels in Dp/CMC mice were higher than those in control/CMC mice; administration of IMD-4690 significantly decreased the production of these interleukins (Fig. 4C-4E). Th1-cytokines such as IFN-γ and IL-12, and TNF-α were also elevated in Dp/CMC mice as compared with control/CMC mice, but IMD-4690 inhibited the production of IFN-γ and TNF-α, but not IL-12 (Fig. 4F-4H).

### Pathological features of airway remodeling in chronic allergen exposure model of bronchial asthma in mice

**Fig 5:**
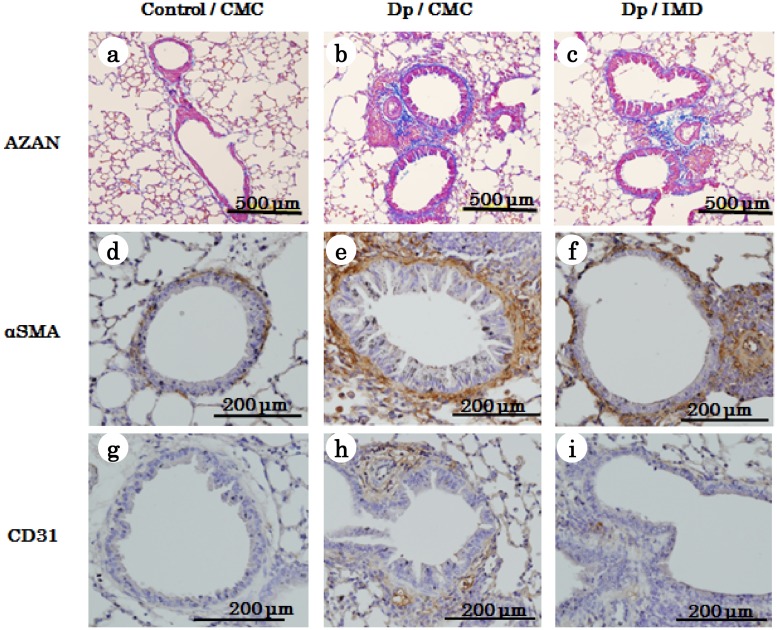
Histopathology of airway remodeling. Photographs are representative of six mice per group. (a-c) Azan–Mallory sections. Original magnification in all figures: ×100. Scale bars in all figures = 500 μm. (d-f) α-SMA immunohistochemistry sections. Original magnification in all figures: ×200. Scale bars in all figures = 200 μm. (g-i) CD31 immunohistochemistry sections. Original magnification in all figures: ×200. Scale bars in all figures = 200 μm.

**Fig 6:**
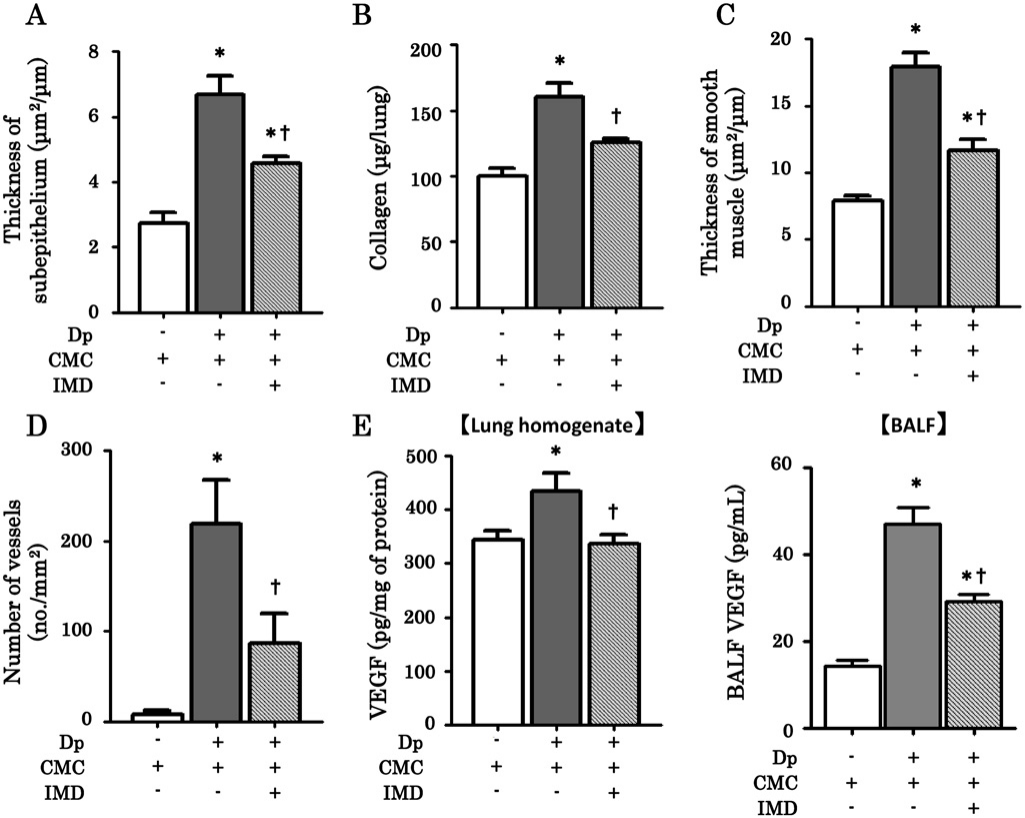
Effect of IMD-4690 on the airway remodeling. (A) Morphological analysis of the thickness of subephithelium. (B) Collagen content in the lungs. The collagen content in the right lung was measured using a Sircol collagen kit. (C-D) Morphological analysis of the thickness of smooth muscle and the CD31 positive blood vessels. (E) VEGF concentration in lung homogenates and BALF. VEGF was measured by ELISA. Data are expressed as means ± SE of six mice. *P < 0.05 compared with control/CMC mice, ^†^P < 0.05 compared with Dp/CMC mice.

To analyze the effects of IMD-4690 on subepithelial fibrosis, we histologically examined subepithelial fibrosis and collagen deposition. In Azan-Mallory-stained sections, subepithelial fibrosis in Dp/CMC mice was increased compared with control/CMC mice (Fig. 5). Subepithelial thickness was significantly decreased by IMD-4690 administration in Dp/IMD mice (Fig. 6A). Similarly, the increased total collagen in the lungs of Dp/CMC mice was reduced by IMD-4690 treatment (Fig. 6B).

We also performed immunohistochemical staining of lung sections with an anti-α-SMA antibody to assess the thickened subepithelial smooth muscle. In the Dp/CMC mice, α-SMA expression was abundant compared with control/CMC mice (Fig. 5). Morphometric analysis showed that IMD-4690 led to a significant reduction in subepithelial smooth muscle thickness (Fig. 6C).

Next, we stained lung sections for CD31 expression to assess airway angiogenesis. Vascular density was markedly increased in Dp/CMC mice compared with control/CMC mice (Fig. 5). The morphometric analysis showed that treatment with IMD-4690 significantly decreased vascular density (Fig. 6D). We also measured VEGF concentrations in lung homogenates and BALF by ELISA. VEGF concentration was increased in Dp/CMC mice compared with control/CMC mice, and treatment with IMD-4690 significantly reduced its concentration (Fig. 6E).

### Effect of IMD-4690 on the levels of mediators related with airway remodeling

**Fig 7:**
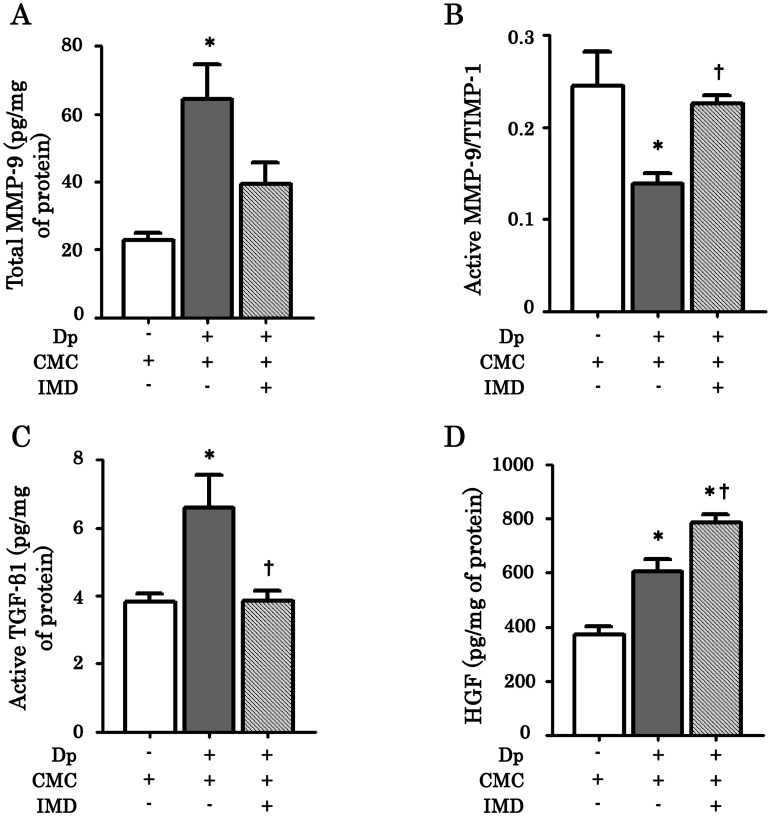
Airway remodeling-related mediators and enzymes in lung homogenates. Frozen lung tissue were solubilized by homogenization in EDTA-free lysis buffer containing protease inhibitors. Samples of the homogenate were centrifuged, and the supernatants were stored at -80°C. (A) The level of total MMP-9. MMP-9 was measured by ELISA. (B) The ratio of active MMP-9/TIMP-1. The amount of active MMP-9 was measured by a Biotrak activity assay kit. (C) The level of active TGF-β1. The level of active TGF-β1 was measured by ELISA. (D) The level of HGF. The level of HGF was measured by ELISA. Data are expressed as means ± SE of six mice. *P < 0.05 compared with control/CMC mice, ^†^P < 0.05 compared with Dp/CMC mice.

Remodeling-related mediators were measured in lung homogenates. Dp exposure significantly increased total MMP-9 production (Fig. 7A). IMD-4690 tended to decrease the total MMP-9 levels, although no significant differences were observed. The ratio of active MMP-9/TIMP-1 was significantly restored by IMD-4690 administration (Fig. 7B). The active form of TGF-β1 in lung homogenates was increased in Dp/CMC mice, and IMD-4690 significantly reduced it (Fig. 7C). Dp/CMC mice showed a marked increase in HGF production compared with the control/CMC mice. In Dp/IMD mice, HGF levels were significantly higher than those in Dp/CMC mice (Fig. 7D).

## Discussion

In the present study, we demonstrated that a novel PAI-1 inhibitor, IMD-4690, effectively ameliorated AHR by reducing airway allergic inflammation and cytokine production such as IL-5 and IL-13 production. In addition, IMD-4690 strongly inhibited airway remodeling by regulating the production of remodeling-related mediators (i.e., TGF-β, VEGF, and HGF) and enzymes (i.e., MMP9) in a chronic antigen exposure mouse model of asthma with Dp.

PAI-1 is secreted as an active form and complexed with either a PA or vitronectin, which is related to the stabilization of active PAI-1 [18–20]. The active PAI-1 spontaneously converts to an inactive form (latent form) [21]. IMD-4690 reduced the level of active PAI-1 and the ratio of active PAI-1/total PAI-1. These results indicate that IMD-4690 may have the potential to reduce the production of PAI-1 as well as to convert it from an active form to an inactive form. In fact, IMD-4690 could inhibit the production of PAI-1 since the total PAI-1 is also reduced by IMD-4690 treatment (data not shown). In addition, IMD-4690 might accelerate the conversion from active to inactive form of PAI-1 via inhibiting the binding of tPA and PAI-1.

Importantly, we also demonstrated that IMD-4690 inhibited airway remodeling via reducing the productions of Th2-cytokines including IL-4, IL-5 and IL-13 in the lungs. Sejima et al. reported that the splenocytes from PAI-1-deficient mice showed the reduced productions of IL-4 and IL-5, and the enhanced production of IFN-γ by OVA stimulation *in vitro* [22]. In the present study, the expression of IFN-γ was not enhanced, but rather slightly inhibited by treatment with IMD-4690, whereas the suppression of Th2 cytokines were prominent. Although the direct interaction between Th2 cytokines and PAI-1 activity in splenocytes are still unclear, the inhibitory effects of IMD-4690 on the expression of Th2 cytokines could exist in the upstream from various activities relating with airway remodeling. In fact, previous studies reported that the absence or knockdown of PAI-1 decreased eosinophilic airway inflammation, AHR, and airway remodeling in the murine model of acute asthma by inducing fibrinolytic responses through plasmin and MMP-9 activations [3, 9, 23]. As suggested in a previous study, although MMP-mediated degradation of ECM proteins leads to improvements in subepithelial fibrosis, the final balance of active MMP-9 to TIMP-1 could be of greatest importance [24]. In fact, IMD-4690 showed the potency to enhance the fibrinolytic response because the active MMP-9/TIMP-1 ratio was elevated by treatment with IMD-4690.

Next, we found that IMD-4690 elevated HGF production. HGF activation is associated with allergic airway inflammation [25] or the antigen-presenting capacity of dendritic cells [26]. HGF is secreted as a single chain protein (scHGF) that is converted to a two-chain heterodimeric active form (tcHGF). uPA can generate active tcHGF from scHGF [27]. uPA and PAI-1 are both up-regulated by allergen exposure in the airway of asthma patients, whereas the inhibitory potential of PAI-1 exceeds the uPA activity [28]. In a PAI-1 deficient murine asthma model, uPA activity was significantly increased. These findings suggest that PAI-1 inhibition is a critical step to regulate the uPA-HGF pathway in allergic airway inflammation. In our model, therefore, HGF seemed to be elevated effectively by the inhibition of PAI-1.

PAI-1 suppression may also inhibit neovascularization by affecting VEGF levels. Previous reports have shown that the absence of PAI-1 attenuates not only the angiogenic response but also VEGF expression [12, 29, 30]. In our study, VEGF levels in lung homogenates and angiogenesis of mice exposed to Dp were decreased by IMD-4690 treatment. VEGF is synthesized by alveolar epithelial cells, bronchial epithelial cells, smooth muscle cells, alveolar macrophages, mast cells, and basophils [31, 32]. Th2 cytokines enhanced VEGF production in the airway [33], whereas VEGF enhances pulmonary Th2 inflammation, remodeling, and angiogenesis [10]. The increase in the number and size of vessels can contribute to thickening of the airway wall, leading to an amplification of bronchial hyperresponsiveness [32]. Moreover, VEGF inhibition attenuates airway inflammation, AHR, and peribronchial fibrosis [10, 34]. These findings suggest that an anti-inflammatory effect of IMD-4690 may result from VEGF suppression.

Furthermore, we noted that administration of IMD-4690 significantly decreased TGF-β in the lung homogenates of mice. TGF-β, enhanced by Th2-inflammatory mediators, plays an important role in airway remodeling, including subepithelial fibrosis and proliferation of airway smooth muscle cells [34–37]. In an allergic airway inflammation model of mice, TGF-β levels were inhibited by treatment with exogenous HGF [25] or VEGF inhibitors [34]. Thus, it is likely that the suppression of airway remodeling by IMD-4690 is associated with synergistic mechanisms, affecting the production of TGF-β, HGF, and VEGF.

Recently, Lee SH et al. [38] showed that a specific PAI-1 inhibitor, tiplaxtinin, attenuated allergic airway inflammation and lung collagen deposition, although the mechanism has not been clearly elucidated. The present study firstly showed the detailed mechanisms involved in the inhibition of airway inflammation and remodeling by a PAI-1 inhibitor with the other compound IMD-4690.

In summary, the present findings strongly indicate that PAI-1 may play an important role for airway inflammation and remodeling of asthma, and that an inhibition of PAI-1 by using IMD-4690 may have therapeutic potential for patients with refractory asthma due to airway remodeling. Because we could not find any adverse effects of IMD-4690 in the mouse model, further study to clarify the effects of IMD-4690 in asthmatic patients would be expected.
